# First case of recent retroviral infection after accidental occupational non-medical exposure to contaminated HIV blood in National Institute for Infectious Diseases "Prof. Dr. Matei Balş" Bucharest, Romania

**DOI:** 10.1186/1471-2334-14-S4-O2

**Published:** 2014-05-29

**Authors:** Elisabeta Otilia Benea, Cozmina Andrei, Georgeta Ducu, Șerban Benea, Dan Oțelea, Sorin Petrea, Manuela Podani, Alina Cozma, Ruxandra Moroti

**Affiliations:** 1 National Institute for Infectious Diseases “Prof. Dr. Matei Balş”, Bucharest, Romania; 2 Carol Davila University of Medicine and Pharmacy, Bucharest, Romania

## 

In 2013, in the National Institute for Infectious Diseases "Prof. Dr. Matei Balş" Bucharest has been applied post-exposure guidelines in 112 cases of occupational exposure to blood and other biological products and was not registered any cases of HIV transmission.

A 22 year old, young police-officer presented in our service for human bite wounds, resulting from intervention in a case of intrafamilial aggression. The aggressor, was known as drug-addicted, co-infected HCV/HIV and he had bleeding in the mouth. He was under antiretroviral therapy for about nine months (CBV+NVP, viral load = 90.787 copies/mL, CD4 = 200 cells/cmm, HCV-RNA = 6,718,917 IU/mL). Exposed person comes to our service in the first two hours of the incident, without performing initial wound toilet. We started antiretroviral prophylaxis (ARVP) with CBV+LPV/r.

At eight days after ARVP, the exposed-person developed a generalized maculopapular rash; in the absence of other clinical manifestations the eruption is interpreted as post-drug side effects. The young man returned to our clinic after another three days (range in which in his own initiative stopped prophylaxis), and we decided to change the antiretroviral regimen with DRV/r + TDF + FTC. Reevaluation after a month recorded that he remained asymptomatic and fourth generation ELISA tests were negative. Reassessment at 12 weeks showed serological tests positive (ELISA, Western blot -p24 = + +), indicating a recent retroviral infection. We find the same HIV sub-type (F) and the same resistance mutations in those two patients, confirming HIV infection to policeman from the aggressor. New patient initially had HIV-RNA = 210,977 copies/mL, CD4 = 789 cells/cmm and was included in the cohort of individuals with recent HIV infection; antiretroviral therapy is initiated with TDF + DRV/r + RAL. After a month of treatment the HIV-RNA = 161 copies/mL blood, HIV-RNA from CFS was undetectable and anti-HCV were negative.

It is the first case of accidental post-exposure (medical and non-medical) to contaminated blood HIV infection in which we applied post-exposure protocol.

It illustrates:

- The importance of adherence to antiretroviral medication;

- The consideration of acute retroviral syndrome in the presence of a maculopapular rash;

- The importance of choosing prophylactic regimen when we know treatment history and possible resistance mutations if the source person is known to be HIV positive.

- Should be monitored more frequently and more complete (HIV-RNA), including people with occupational non-medical exposure.

